# Changes in Pre- and Post-Exercise Gene Expression among Patients with Chronic Kidney Disease and Kidney Transplant Recipients

**DOI:** 10.1371/journal.pone.0160327

**Published:** 2016-08-12

**Authors:** Dawn K. Coletta, Latoya E. Campbell, Jennifer Weil, Bruce Kaplan, Marie Clarkson, Jean Finlayson, Lawrence J. Mandarino, Harini A. Chakkera

**Affiliations:** 1 Division of Nephrology and Hypertension, Mayo Clinic, Phoenix, Arizona, United States of America; 2 School of Life Sciences, Arizona State University, Phoenix, Arizona, United States of America; 3 School for the Science of Health Care Delivery, Arizona State University, Phoenix, Arizona, United States of America; 4 Mayo Clinic in Arizona, Scottsdale, Arizona, United States of America; 5 Department of Basic Medical Sciences, University of Arizona College of Medicine-Phoenix, Phoenix, Arizona, United States of America; 6 NIH NIDDK, Phoenix, Arizona, United States of America; The University of Tokyo, JAPAN

## Abstract

**Introduction:**

Decreased insulin sensitivity blunts the normal increase in gene expression from skeletal muscle after exercise. In addition, chronic inflammation decreases insulin sensitivity. Chronic kidney disease (CKD) is an inflammatory state. How CKD and, subsequently, kidney transplantation affects skeletal muscle gene expression after exercise are unknown.

**Methods:**

Study cohort: non-diabetic male/female 4/1, age 52±2 years, with end-stage CKD who underwent successful kidney transplantation. The following were measured both pre-transplant and post-transplant and compared to normals: Inflammatory markers, euglycemic insulin clamp studies determine insulin sensitivity, and skeletal muscle biopsies performed before and within 30 minutes after an acute exercise protocol. Microarray analyses were performed on the skeletal muscle using the 4x44K Whole Human Genome Microarrays. Since nuclear factor of activated T cells (*NFAT*) plays an important role in T cell activation and calcineurin inhibitors are mainstay immunosuppression, calcineurin/*NFAT* pathway gene expression was compared at rest and after exercise. Log transformation was performed to prevent skewing of data and regression analyses comparing measures pre- and post-transplant performed.

**Result:**

Markers of inflammation significantly improved post-transplantation. Insulin infusion raised glucose disposal slightly lower post-transplant compared to pre-transplant, but not significantly, thus concluding differences in insulin sensitivity were similar. The overall pattern of gene expression in response to exercise was reduced both pre-and post-transplant compared to healthy volunteers. Although significant changes were observed among *NFAT*/Calcineurin gene at rest and after exercise in normal cohort, there were no significant differences comparing *NFAT*/calcineurin pathway gene expression pre- and post-transplant.

**Conclusions:**

Despite an improvement in serum inflammatory markers, no significant differences in glucose disposal were observed post-transplant. The reduced skeletal muscle gene expression, including *NFAT*/calcineurin gene expression, in response to a single bout of exercise was not improved post-transplant. This study suggests that the improvements in inflammatory mediators post-transplant are unrelated to changes of *NFAT*/calcineurin gene expression.

## Introduction

Normal insulin sensitivity is associated with increased gene expression from skeletal muscle after exercise [1]. A decrease in insulin sensitivity is associated with decreased gene expression from skeletal muscle [2, 3]. Chronic kidney disease (CKD) is marked by decrease in insulin sensitivity [4, 5]. Whether reduced gene expression in skeletal muscle in response to exercise extends to people with CKD is not known. In addition, transplant is associated with a lowered inflammatory milieu, whether this decrease is associated with genes related to the immunosuppressive medications given to these patients is also not known.

Additionally, chronic inflammatory states promote insulin resistance and CKD is a chronic inflammatory state [6, 7]. After transplant, inflammation may be reduced both by the addition of a new kidney and by the immunosuppressive medication that is administered. Successful kidney transplantation is the best therapy for patients with end-stage CKD [8]; however, there is a reported 20–30% increased risk in development of new-onset diabetes after transplantation in the first year after transplant [9, 10]. The rapid development of new onset diabetes after transplantation (NODAT) within the first year after transplant is not well understood. The pathogenesis of NODAT is multifactorial including a component of insulin resistance [11]. Commonly used immunosuppression including glucocorticoids and calcineurin inhibitors have been recognized as contributing to insulin resistance and play a role in the development of NODAT [12, 13].

This study compares markers of inflammation, measures of glucose homeostasis and skeletal muscle gene expression at rest and after a bout of exercise among a cohort of patients with end-stage renal disease before and after a successful kidney transplant.

## Concise Methods

### Study cohort

After approval from the Mayo Clinic Institutional Review Board, we conducted a prospective cohort study of adult, non-diabetic patients scheduled for living donor kidney transplant. Absence of diabetes and impaired glucose tolerance was documented with a normal 2-hour oral glucose tolerance test (OGTT) based on the American Diabetes Association (ADA) criteria. Additionally, all patients had screening fasting blood glucose <126 mg/dl and HbA1C <6.5%. Study patients included only those who were at low immunologic risk for rejection based on their pre-transplant cross match testing, thus they were all on a rapid steroid taper immunosuppression protocol. The rapid steroid taper immunosuppression protocol includes that all patients receive a 5-day tapering course of glucocorticoids (methylprednisolone intravenously 500 mg on day 1; 250 mg on day 2; 125 mg on day 3; oral prednisone 60 mg on day 4; and 30 mg on day 5; then discontinued). Maintenance immunosuppression after transplant included tacrolimus (a calcineurin inhibitor) and mycophenolate mofetil.

### Experimental protocol

**Fig 1:**
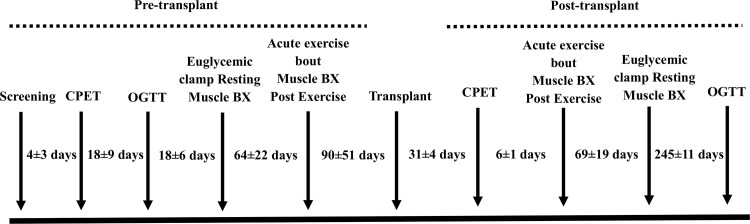
Overall design of study.

The overall study design is shown in Fig 1. Written consent was obtained from study participants at least 6 weeks prior to the date of kidney transplant. They reported to the Clinical Studies Infusion Unit at Mayo Clinic in Arizona after an overnight fast and underwent screening laboratory tests including a complete blood count, serum creatinine, HbA1C, lipid panel, and cardiopulmonary exercise testing (CPET) to estimate maximum watts at peak oxygen uptake or VO_2peak_ was performed. One to 3 weeks later, participants returned to the clinic for the 2-hour OGTT. Subsequently, a euglycemic hyperinsulinemic clamp with peripheral skeletal muscle biopsy was performed at baseline and at the end of the study as described in Fig 1. After the clamp (both at baseline and after the transplant), the patient underwent ergometry test on the bicycle with goal to reach 90% of target watts and at the end of which a peripheral skeletal muscle biopsy was performed. The same set of tests, including ergometry tests and skeletal muscle biopsy, was performed after 1 month post-transplant. All patients were instructed to not engage in voluntary exercise for 3 days before each study visit [3].

### Euglycemic clamp with muscle biopsy

A 2-hour euglycemic clamp using an insulin infusion rate of 80 mU^.^m^-2.^min^-1^ was performed as previously described [14, 15]. A primed infusion of 6,6 di-deuterated glucose was started at -120 minutes to determine the basal rate of glucose metabolism. Sixty minutes after the start of deuterated glucose, a basal *vastus lateralis* muscle biopsy was performed. After 60 minutes, an insulin infusion was started. Fifteen minutes into the insulin infusion, the deuterated glucose was discontinued. A variable infusion of 20% dextrose enriched with 6,6 di-deuterated glucose was used to maintain euglycemia and a constant enrichment of the tracer. Enrichment of plasma glucose with 6,6 di-deuterated glucose was measured by the Center for Clinical and Translational Science Metabolomics Core at the Mayo Clinic. The rates of glucose appearance and disappearance were calculated using steady state equations.

### Exercise testing

Patients underwent exercise test using a cycle ergometer. Criteria for end of the test were no further increases in heart rate and a respiratory exchange ratio greater than 1.1. From the VO_2peak_ test, heart rates at 70 and 90 percent of maximum effort were determined. The acute exercise bout experiment was designed to elicit gene expression responses in muscle and consisted of 4 sets of exercise, separated by 2 minutes of rest (unloaded pedaling). Patients exercised in each set at 70% of maximum effort for 8 minutes, followed by a 2 minute “sprint” at 90% of maximum effort, with the 2-minute rest interval following. This was repeated 4 times, for a total of 48 minutes of exercise. The design of the acute exercise bout was identical to one used previously for healthy volunteers [3].

### Muscle biopsy processing and mRNA analysis

RNA was isolated from the *vastus lateralis* muscle biopsies as previously described [16]. Microarray analysis was performed using the 4x44K Whole Human Genome Microarrays (Agilent Technologies, Palo Alto, CA) as previously described [3]. Data were deposited in the Gene Expression Omnibus (http://www.ncbi.nlm.nih.gov/geo/index.cgi), submission number GSE68585. To assess the extent to which pre- and post-transplant exercise-induced gene expression responses differed from responses in healthy control subjects who performed an identical exercise protocol [3], we performed regression analysis comparing our subjects to normal controls.

### Other analyses

Plasma glucose concentration was determined by the glucose oxidase method on an YSI 2300 STAT plus (YSI INC., Yellow Springs, OH). Plasma insulin was measured by a two-site immunoenzymatic assay performed on the DxI 800 automated immunoassay system (Beckman Instruments, Chaska, MN 55318). Comprehensive metabolic, lipid, cytokine and hemogram panels were performed by the Biospecimens Accessioning and Processing Core at the Mayo Clinic in Arizona on pre-transplant, 1 month, 4 months, and 12 months post-transplant fasting samples. To assess potential changes in inflammation markers, plasma cytokine concentrations were assayed before and after kidney transplant.

### Analytical methods

Glucose and insulin areas under the glucose tolerance curve were calculated by the trapezoid method. Disposition index was calculated as the change in glucose compared to the change in insulin over the first 30 minutes of the glucose tolerance test. The statistical significance of difference between means for *in vivo* data was determined using paired or non-paired Student’s t-tests where appropriate. Pearson correlation calculation was used for all correlations presented.

## Results

### Patient characteristics and inflammatory markers pre- and post-transplant

Four men and one woman, all of whom were non-diabetic, had end-stage CKD and were undergoing dialysis, participated in the study (Table 1). Patients before transplant were overweight, and body mass index increased slightly but not significantly after transplant. Before transplant, the patients had elevated triglyceride concentrations, but normal plasma insulin, glucose, and HbA1c levels. HbA1c levels increased after transplant.

**Table 1:** Patient characteristics and inflammatory markers pre- and post-transplant.

	Pre-Transplant	1 Month Post-Transplant	4 Months Post-Transplant	12 Months Post-Transplant
Age (y)	52 ± 2			
Gender (M/F)	4/1			
BMI (kg·m^-2^)	26.4 ± 0.8	26.5 ± 0.8	27.1 ± 0.7	28.0 ± 0.6
Triglycerides (mg/dL)	163 ± 20	222 ± 46	204 ± 29	163 ± 19
Total cholesterol (mg/dL)	188 ± 10	203 ± 13	189 ± 13	153 ± 21
Fasting glucose (mM)	5.20 ± 0.22	5.70 ± 0.16	5.64 ± 0.17	5.61 ± 0.23
Fasting plasma insulin (pM)	47 ± 7	62 ± 13	N.D.	N.D.
Creatinine (mg/dL)*	3.26 ± 0.5	1.60 ± 0.1	1.4 ± 0.1	1.52 ±0 .1
Hemoglobin (g/dL)**	11.8 ± 0.6	12.1 ± 0.5	12.7 ± 0.8	13.1 ± 0.9
Hematocrit (%)**	34.7 ± 1.9	36.1 ± 1.4	38.2 ± 2.3	38.9 ± 2.5
HbA1c (%)*	5.4 ± 0.1	5.4 ± 0.1	5.5 ± 0.1	5.7 ± 0.1
IL-2 (pg/mL)	0.48 ± 0.02		0.41 ± 0.04	
IL-8 (pg/mL)	11.9 ± 5.2		7.8 ± 1.8	
IL-12 (pg/mL)	0.51 ± 0.12		0.65 ± 0.15	
IL-1b (pg/mL)	0.29 ± 0.03		0.35 ± 0.05	
IL-6 (pg/mL)	1.0 ± 0.2		1.1 ± 0.2	
IL-10 (pg/mL)	1.1 ± 0.3		1.8 ± 0.4*	
GM-CSF (pg/mL)	23.2 ± 2.3		21.1 ± 4.6	
Interferon 0γ (pg/mL)	1.4 ± 0.3		1.4 ± 0.2	
Tumor necrosis factor α (pg/mL)	3.6 ± 0.2		2.2 ± 0.3*	
Adiponectin (ng/mL)	9835 ± 2961		7101 ± 2182	
Leptin (pg/mL)	18.5 ± 10.2		9.4 ± 3.6	

Values are given as mean ± SEM. *P < 0.05 versus pre-transplant values, paired t-test. Data are shown as mean ± SEM.

*P < 0.05

**P < 0.01, significant difference among means using repeated measures analysis of variance.

Plasma IL-10 concentrations increased significantly and plasma TNFα concentrations decreased significantly after transplant. Other cytokines including leptin and adiponectin demonstrated decrease although not statistically significant.

### Exercise characteristics pre- and post-transplant

The CPET was performed pre-transplant and post-transplant and results were compared. Although all patients reported a level of physical activity above the sedentary level, VO_2peak_ values before and after transplant were low (Table 2).

**Table 2:** Exercise characteristics of patients.

	Pre-Transplant	Post-Transplant*
*Maximal Exercise Test*		
VO_2peak_ (ml·kg^-1^·min^-1^)	19.9 ± 2.7	18.7 ± 2.1
Work_peak_ (Watts)	149 ± 18	136 ± 25
Heart rate peak (BPM)	130 ± 10	125 ± 7
*Acute Exercise Bout for gene expression*		
Predicted 70% heart rate (BPM)	91 ± 7	87 ± 6
Actual 70% heart rate (BPM)	99 ± 6	93 ± 9
Predicted 90% heart rate (BPM)	117 ± 9	112 ± 8
Actual 90% heart rate (BPM)	120 ± 7	131 ± 11

Data are given as mean ± SEM.

*Exercise test performed on average 31 days post-surgery

Moreover, peak heart rates during the maximal exercise test were far below age-adjusted predicted maximum heart rates. These variables did not improve following kidney transplant. Nevertheless, during the acute exercise bout designed to assess gene expression changes in muscle, all patients were able to successfully complete the 70% and 90% periods of exercise, repeated 4 times, and this was the case both before and after kidney transplant.

### Glucose metabolism and insulin sensitivity pre- and post-transplant

There was no significant difference in glucose tolerance as a result of kidney transplantation. Although glucose tolerance was somewhat reduced after transplant, neither mean glucose (193 ± 48 pre vs. 316 ± 102 mM●min post-transplant) nor mean insulin excursion (20.2 ± 4.9 pre vs. 28.0 ± 5.5 nM●min post-transplant (areas under the glucose tolerance curve) were significantly higher post-transplant. Mean disposition index was 137 ± 8 pre-transplant and 390 ± 264 mM●pM^-1^ post-transplant. The large variability was due to 1 patient, and when this value was excluded, post-transplant mean disposition index post-transplant was 127 ± 29 mM●pM^-1^. There also was no difference in mean Δinsulin _(30–0)_, which was 345 ± 75 and 355 ± 46 pM pre-and post-transplant, respectively.

**Fig 2:**
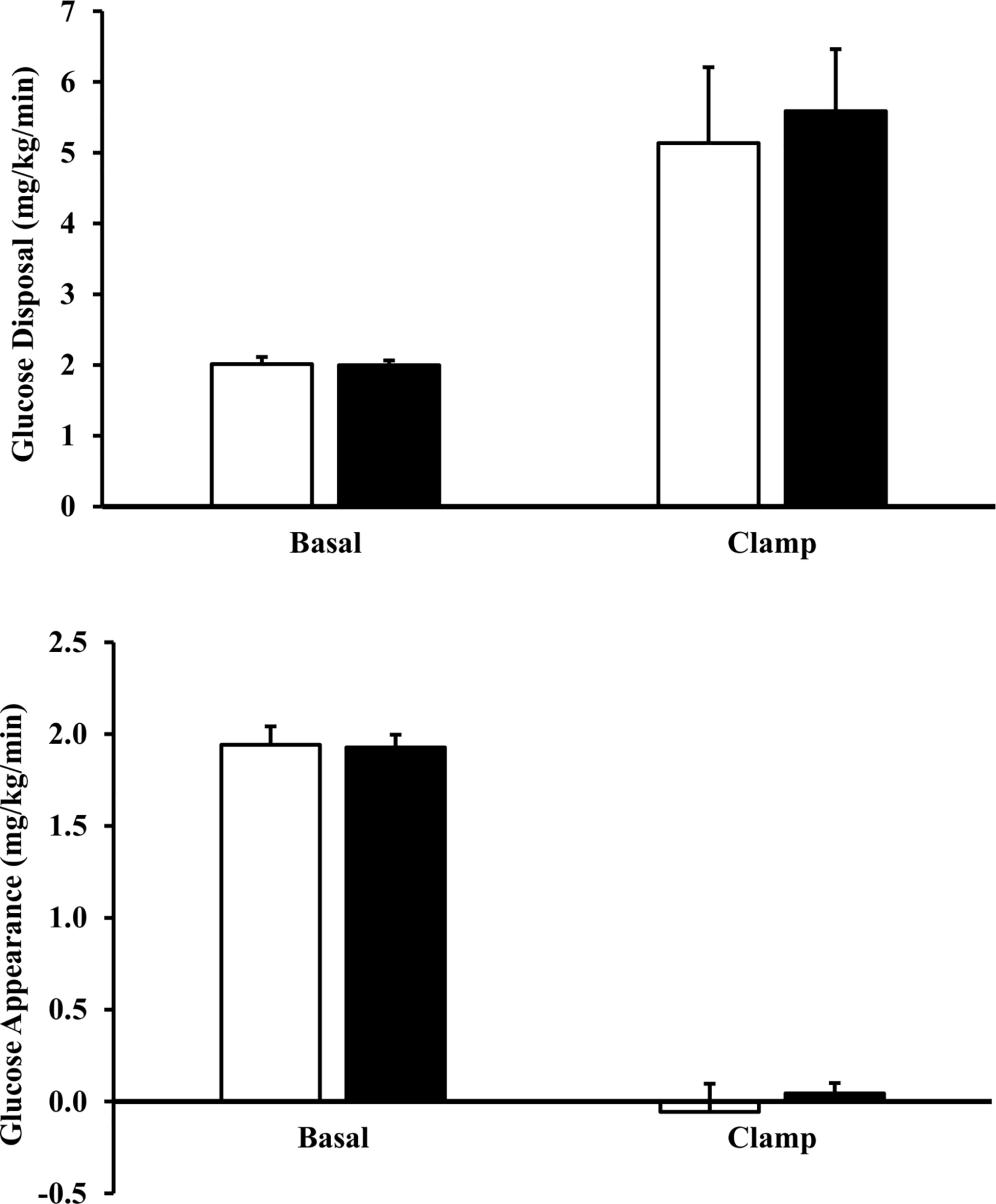
Glucose metabolism in the post-absorptive state and after two hours of insulin infusion in kidney transplant patients (n = 5) pre-transplant (open bars) and post-transplant (closed bars). Rates of basal and insulin-stimulated glucose disposal are given in panel A, and rates of basal and insulin-suppressed endogenous glucose production (B) were determined using 6,6 di-deuterated glucose. An 80 mU/(m^2^●min) insulin infusion was used. Data are shown as means ± SEM.

There was no change in insulin sensitivity among the 5 subjects post-transplant. Mean levels of plasma insulin achieved during the last 30 minutes of the 2-hour insulin infusion were similar before and after transplant (775 ± 48 vs. 789 ± 58 pM, pre- vs. post-transplant, respectively). All patients had normal post-absorptive levels of endogenous glucose production before and after transplant. Glucose production was suppressed completely before and after transplant by insulin (Fig 2). Insulin infusion raised glucose disposal to a similar extent before and after transplant.

### Gene expression in skeletal muscle at rest and after a bout of exercise before and after kidney transplant

#### Effect of exercise on gene expression in muscle before kidney transplant

Percutaneous muscle biopsies were obtained at rest and 30 minutes after completing a 48-minute stationary cycle exercise bout in patients before kidney transplant. There were 1693 probes that changed by 1.5-fold after exercise, with nominal P ≤ 0.05 (data not shown). The 1693 probes were subjected to Kyoto Encyclopedia of Genes and Genomes (KEGG) pathway and gene ontology annotation analysis using the Database for Annotation, Visualization and Integrated Discovery (DAVID) v6.7. The most significant pathways are shown in S1 Table. Significantly (Benjamini-Hochberg corrected P < 0.05) enriched KEGG pathways included cytokine-cytokine receptor interaction, focal adhesion, extracellular matrix-receptor interaction, and *TGFβ* signaling (S1 Table). Significant gene ontology (GO) pathways (molecular function) included heparin binding, actin binding, and extracellular matrix structural constituent, among others (S1 Table).

#### Gene expression in skeletal muscle after kidney transplant at rest

Agilent 4x44K whole human genome microarrays were used to evaluate differences in basal, resting gene expression levels in patients before and after kidney transplant. A total of 612 microarray probes were altered (increased or decreased) greater than 1.5 fold with a nominal P value ≤0.05 (data not shown). Among the 10 probes with the greatest fold increases in expression after transplant, 4 were for collagen subunits, including *COL1A1*, *COL6A6*, *COL3A1* (2 probes) and another was for an integrin binding protein (*ITG1BP3* or *NRK2*). The 612 probes were subjected to KEGG pathway and GO annotation analysis using DAVID. Significantly (Benjamini-Hochberg corrected P < 0.05) enriched KEGG pathways included extracellular matrix-receptor interaction, focal adhesion, and *TGFβ* signaling (S1 Table). Significant GO pathways (molecular function) included extracellular matrix, growth factor binding, calcium ion binding, and *PDGF* binding.

#### Gene expression in skeletal muscle after kidney transplant after a bout of exercise

One month after kidney transplant, patients had a repeat acute exercise bout with muscle biopsies at rest and 30 minutes after the conclusion of exercise. Microarray analysis revealed 1176 genes that increased or decreased 1.5-fold with a nominal P value of 0.05 or better. A total of 1176 microarray probes were altered (increased or decreased) greater than 1.5 fold with a nominal P value ≤0.05. The 1176 probes were subjected to KEGG and GO annotation analyses using DAVID v6.7. The most significant pathways are shown in S1 Table.

**Fig 3:**
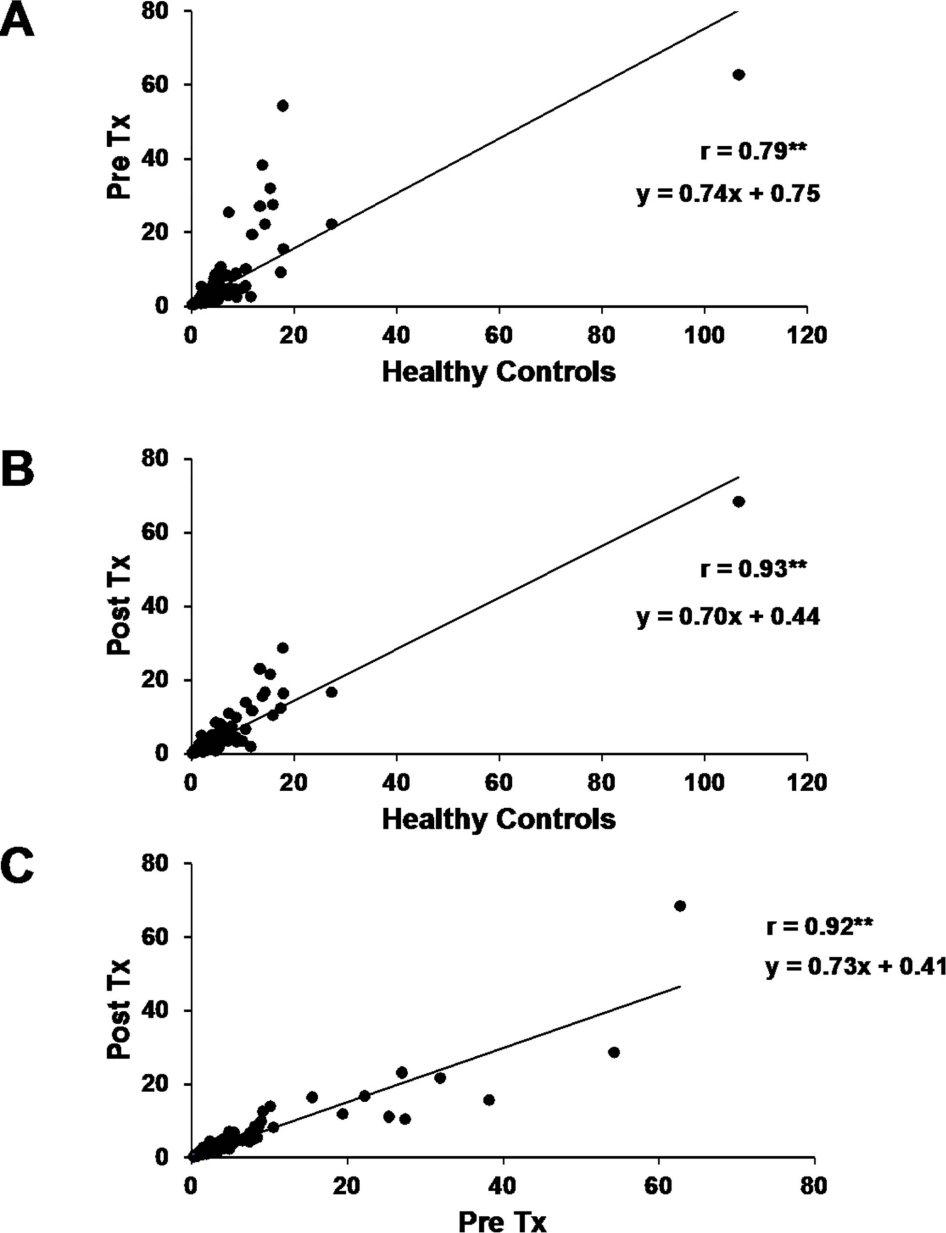
Relationships between exercise-induced gene expression changes (delta from basal, resting gene expression) for (A) pre-transplant patients vs. healthy controls, (B) post-transplant patients vs. healthy controls, and (C) post vs. pre-transplant effects of exercise. P < 0.01 for correlation. Data were expressed as differences between exercise-induced fold-changes in gene expression in each of the three comparisons.

Changes in response to exercise were compared with normative data from the 301 genes that changed significantly in healthy subjects [3]. Results of these regression analyses are shown in Fig 3. Both pre- and post-transplant changes were highly correlated with normative data (Fig 3A and 3B, respectively, r = 0.79 and 0.93, respectively, P < 0.01). However, the regression coefficients relating normal responses to pre- and post-kidney transplant responses were 0.74 and 0.70 respectively, which were significantly lower than a value of 1.0 that would have been expected had results been identical (P < 0.05).To gauge whether post-transplant exercise responses were altered compared to pre-transplant values, exercise-induced changes in gene expression were compared by regression analysis and demonstrated high correlation (Fig 3C, r = 0.92, P < 0.01).

#### Effect of transplant on resting and after exercise gene expression involved in calcineurin/*NFAT* signaling

To determine whether tacrolimus (calcineurin inhibitor) after kidney transplant could alter exercise-induced changes in genes that are part of the calcineurin/*NFAT* signaling (http://www.broadinstitute.org/gsea/msigdb/index.jsp), genes annotated to this pathway were compared for exercise responses. Results showed that overall, among all 178 genes in this pathway, there was no significant response to exercise in healthy controls or participants pre- or post-transplant (data not shown). Examining the 7 genes that increased by at least 1.5 fold after exercise in healthy controls (2.68 ± 0.74 fold, average) these also were increased in the pre- and post-transplant condition (2.35 ± 0.42 and 2.51 ± 0.41 fold, respectively) (Table 3).

**Table 3:** *NFAT* Gene Expression.

Gene Symbol	Healthy controls fold change	Healthy controls P value	Pre-transplant fold change	Pre-transplant P value	Comparing Pre-transplant fold change to healthy fold change (P value)	Post-transplant fold change	Post-transplant P value	Comparing post-transplant fold change to healthy fold change P value	Comparing pre-transplant fold change to post-transplant fold change (P value)
*CDKN1A*	1.56	0.00015	3.65	0.063	0.053806558	3.56	0.0025	0.000772708	0.972211502
*NPPA*	1.61	0.035	0.86	0.155	0.152388407	1.3	0.29	0.664144399	0.348294588
*CDKN1A*	1.76	4.6E-06	2.42	0.048	0.252030417	2.38	0.022	0.180687496	0.978413125
*EDN1*	1.98	0.00038	2.07	0.047	0.895503294	2.33	0.034	0.625909669	0.819630648
*FKBP1A*	2.27	1.29E-06	1.49	0.075	0.066983948	1.52	0.028	0.056927083	0.934217025
*PIM1*	2.56	3.41E-07	2.07	0.021	0.362681298	2.15	0.00021	0.355897099	0.86703075
*NR4A1*	7.02	5.48E-10	3.93	0.0082	0.062345795	4.32	0.000051	0.044233032	0.818356927
*PRKAR2B*	0.55	0.0027	1.03	0.42	0.063167806	0.84	0.298	0.24181481	0.56765799
*PRKCB*	0.59	0.048	0.99	0.49	0.344460401	0.61	0.022	0.964624765	0.172838548
*MAP3K1*	0.61	0.018	0.79	0.095	0.477248431	0.82	0.226	0.436514375	0.868931367
*HAND1*	0.63	0.036	0.95	0.38	0.331765088	0.67	0.084	0.872455832	0.372859202

Examining the 4 calcineurin/*NFAT* signaling that decreased by at least 1.5 fold after exercise in healthy controls (0.59 ± 0.02 fold, average) these did not decrease in the pre-transplant condition (0.94 ± 0.05 fold, average). However, there was a reduction in the expression of these genes post-transplant (0.74 ± 0.06, average) (Table 3).

## Discussion

Chronic kidney disease is a pro-inflammatory and insulin-resistant state. In normal subjects, insulin resistance is associated with abnormal gene expression in skeletal muscle after exercise. Among our study cohort of non-diabetics pre-kidney transplant, after a successful kidney transplant, none of the patients developed NODAT. We observed improvement in inflammatory markers post-transplant but no statistical improvement in insulin sensitivity. Reduced gene expression in skeletal muscle after exercise was present both pre- and post-transplant.

Based on previous reports, we hypothesized that we would see glucose intolerance pre-transplant, and our patients in fact did have moderately lower insulin sensitivity compared to healthy controls [4, 5, 17]. We hypothesized that post-transplant we would see an improvement in glucose sensitivity, but this did not happen. Despite a decrease in inflammatory state post-transplant, insulin sensitivity was not improved. Patients with CKD had evidence of systemic inflammation, suggested by increased plasma leptin and TNFα and decreased adiponectin concentrations, relative to normative data. Post-transplant, a significant decline in plasma adiponectin, leptin, and TNFαwas observed.

We also hypothesized that transplant and immunosuppression would result in decreased gene expression from skeletal muscle after exercise secondary to the insulin resistance that accompanies transplant immunosuppression medications. Among subjects with CKD, gene expression response to exercise was qualitatively similar to that of healthy volunteers. Robust responses were observed for a variety of transcription factors and genes involved in angiogenesis, thrombospondins, and immediate early response genes. Transcription of genes is identical to that of healthy volunteers and this may, in part, explain why physical activity can improve patient outcomes [18]. However, quantitatively the overall gene expression response to exercise in patients with CKD to that in healthy people was lowered by a factor of about 25–30%. Whether this reduced response can be attributed to insulin resistance or to other factors associated with CKD is unknown.

We specifically examined the *NFAT*/calcineurin pathway genes to see if calcineurin inhibitors decreased expression. This is the pathway through which calcineurin inhibits T-cells and blocks an immune response to transplant. We did not observe any significant changes from pre-transplant to post-transplant in the expression of these genes. In other studies, blockade of calcineurin signaling blocks muscle hypertrophy in the overloaded condition [19]. This may be independent of expression of *NFAT*/calcineurin genes [20, 21] and instead might be related to the cellular localization of MEF2C [19]. However, other effects of immunosuppression with calcineurin inhibitors cannot be excluded.

With the DAVID analysis of the resting muscle biopsies before and after transplant (independent to exercise effects), we see an enrichment for inflammatory (TGF-beta signaling pathway), extracellular matrix (ECM-receptor interaction) and metabolic (insulin-like growth factor binding) pathways. DAVID analysis of the significant genes altered by exercise before transplant identified a number of pathways involved in extracellular matrix (ECM-receptor interaction), inflammation (TGF-beta signaling pathway) and cytoskeletal (actin binding) interactions. Previous studies from our lab have demonstrated alterations in genes and proteins coding for extracellular matrix remodeling, inflammation and cytoskeletal interactions in the skeletal muscle of experimental and naturally occurring insulin resistance [16, 22]. These same pathways remained enriched following exercise after transplant with the exception of actin binding. There are significantly more pathways enriched in the post-transplant exercise state compared to the pre-transplant exercise state, and interestingly a large number of these pathways are for cytokine and chemokine activity. Since our patients are in recovery phase post-transplant, it is expected that they are still recovering from their surgery and hence the enrichment for the inflammatory pathways (i.e. cytokine activity and chemokine activity).

Pre-transplant subjects evidenced reduced exercise tolerance with lower than normal values for peak heart rate, work, and peak oxygen consumption during a maximal exercise test. They had low hematocrit and blood hemoglobin values, as expected in CKD, potentially reducing oxygen delivery to tissues during exercise. After transplant, patients showed improvements in a number of areas. As expected, hematocrit and hemoglobin levels improved; however, post-transplant we did not observe an improvement in VO_2peak_. We presume that patients were still recovering from bed rest. Exercise testing and muscle biopsy after exercise were performed on average 31 days after surgery. Bed rest is known to cause decrease in skeletal muscle mass primarily due to decrease in protein synthesis but also because of an increase in proteolytic enzymes. Previous studies of gene expression have shown that both are active in disuse atrophy [23, 24]. The VO_2peak_ has been shown elsewhere to improve 1 year post-transplant [25]. Although there was improvement in inflammatory markers, insulin sensitivity in our subjects remained low-normal post-transplant. Calcineurin inhibitor-based immunosuppression and albeit minimal steroid exposure were probably contributory to low-normal insulin sensitivity [25]. Decreased insulin sensitivity might explain the continued reduced-exercise tolerance as well as decreased gene expression.

In summary, patients with CKD have reduced skeletal muscle gene expression in response to a single bout of exercise which is not changed by improved renal function after transplantation. Despite a significant decrease in inflammatory markers post-transplant, insulin sensitivity, and gene expression in response to exercise including the *NFAT*/calcineurin pathway was not improved post-transplant. Even though this study demonstrates reduced gene expression responses in skeletal muscle of patients with CKD before and after kidney transplant, the mechanism is yet to be defined. The clinical impact of these findings needs to be validated in a larger cohort.

## Supporting Information

S1 Table DAVID analysis of gene expression after (A) one bout of exercise before transplant; (B) rest after transplant; (C) one bout of exercise after transplant.
(DOCX)

S2 Table Gene ontology (GO) molecular function analysis by DAVID on genes after transplant that had at least a 1.5-fold greater expression before transplant. (DOCX)
