# Supplementary material for: Changes in Pre- and Post-Exercise Gene Expression among Patients with Chronic Kidney Disease and Kidney Transplant Recipients
Source: PLoS One. 2016 Aug 12;11(8):e0160327. doi: 10.1371/journal.pone.0160327 (PMC4982681; doi:10.1371/journal.pone.0160327)
Supplement: S1 Table — DAVID analysis of gene expression after (A) one bout of exercise before transplant; (B) rest after transplant; (C) one bout of exercise after transplant. (DOCX) [file pone.0160327.s001.docx]

Supplemental Table 1.

| **Supplemental Table 1. DAVID analysis of gene expression after (A) one bout of exercise before transplant; (B) rest after transplant; (C) one bout of exercise after transplant**. | | | | | |
| --- | --- | --- | --- | --- | --- |
| Term | Gene Count | # Genes Increased | # Genes Decreased | Enrichment | FDR (%) |
|  |  |  |  |  |  |
| **David analysis of genes altered by exercise before transplant** |  |  |  |  |  |
| GO:0008201~heparin binding | 20 | 16 | 4 | 3.65 | 0.003 |
| hsa04060:Cytokine-cytokine receptor interaction | 35 | 28 | 7 | 2.29 | 0.01 |
| GO:0001871~pattern binding | 23 | 16 | 7 | 2.81 | 0.032 |
| GO:0030247~polysaccharide binding | 23 | 16 | 7 | 2.81 | 0.032 |
| hsa05130:Pathogenic Escherichia coli infection | 13 | 13 | 0 | 4.03 | 0.071 |
| GO:0005539~glycosaminoglycan binding | 21 | 16 | 5 | 2.82 | 0.076 |
| hsa04510:Focal adhesion | 26 | 24 | 2 | 2.28 | 0.16 |
| GO:0005201~extracellular matrix structural constituent | 15 | 12 | 3 | 3.28 | 0.25 |
| hsa04512:ECM-receptor interaction | 15 | 15 | 0 | 3.15 | 0.255 |
| GO:0003779~actin binding | 34 | 29 | 5 | 1.96 | 0.44 |
| hsa05416:Viral myocarditis | 13 | 12 | 1 | 3.23 | 0.629 |
| hsa05200:Pathways in cancer | 33 | 32 | 1 | 1.78 | 1.675 |
| GO:0030246~carbohydrate binding | 34 | 22 | 12 | 1.8 | 1.843 |
| GO:0005525~GTP binding | 35 | 23 | 12 | 1.77 | 2.185 |
| GO:0019001~guanyl nucleotide binding | 35 | 23 | 12 | 1.72 | 3.377 |
| GO:0032561~guanyl ribonucleotide binding | 35 | 23 | 12 | 1.72 | 3.377 |
| GO:0005518~collagen binding | 8 | 8 | 0 | 4.18 | 3.805 |
| hsa04350:TGF-beta signaling pathway | 13 | 13 | 0 | 2.64 | 3.821 |
| GO:0016564~transcription repressor activity | 30 | 23 | 7 | 1.78 | 4.418 |

|  |  |  |  |  |  |
| --- | --- | --- | --- | --- | --- |
| **David analysis of genes altered after transplant in resting muscle.** |  |  |  |  |  |
| GO:0005201~extracellular matrix structural constituent | 19 | 19 | 0 | 10.17 | 4.00E-10 |
| hsa04512:ECM-receptor interaction | 17 | 16 | 1 | 9.36 | 1.39E-08 |
| GO:0005509~calcium ion binding | 48 | 36 | 12 | 2.4 | 3.62E-05 |
| hsa04510:Focal adhesion | 20 | 19 | 1 | 4.6 | 3.66E-05 |
| GO:0019838~growth factor binding | 15 | 13 | 3 | 6.58 | 8.16E-05 |
| GO:0048407~platelet-derived growth factor binding | 6 | 6 | 0 | 25.11 | 0.003 |
| GO:0005198~structural molecule activity | 29 | 26 | 3 | 2.11 | 0.392 |
| hsa04350:TGF-beta signaling pathway | 9 | 8 | 1 | 4.78 | 0.552 |
| GO:0008201~heparin binding | 9 | 8 | 1 | 4.02 | 2.509 |
| GO:0005520~insulin-like growth factor binding | 5 | 5 | 1 | 9.21 | 2.71 |
| GO:0005539~glycosaminoglycan binding | 10 | 9 | 1 | 3.29 | 4.807 |

|  |  |  |  |  |  |
| --- | --- | --- | --- | --- | --- |
| **David analysis of genes altered by exercise after transplant** |  |  |  |  |  |
| hsa04060:Cytokine-cytokine receptor interaction | 28 | 20 | 8 | 2.68 | 0.003 |
| GO:0046983~protein dimerization activity | 41 | 30 | 11 | 1.91 | 0.158 |
| GO:0030528~transcription regulator activity | 89 | 60 | 29 | 1.49 | 0.164 |
| GO:0005125~cytokine activity | 19 | 14 | 5 | 2.59 | 0.399 |
| GO:0008009~chemokine activity | 8 | 7 | 1 | 4.94 | 0.588 |
| GO:0042379~chemokine receptor binding | 8 | 7 | 1 | 4.64 | 0.914 |
| hsa04350:TGF-beta signaling pathway | 12 | 10 | 2 | 3.34 | 0.919 |
| hsa04621:NOD-like receptor signaling pathway | 9 | 7 | 2 | 3.91 | 1.012 |
| GO:0016564~transcription repressor activity | 26 | 23 | 3 | 2.08 | 1.151 |
| hsa04062:Chemokine signaling pathway | 17 | 10 | 7 | 2.33 | 1.874 |
| hsa05200:Pathways in cancer | 25 | 18 | 7 | 1.92 | 2.11 |
| GO:0003700~transcription factor activity | 58 | 38 | 20 | 1.5 | 2.625 |
| hsa04060:Cytokine-cytokine receptor interaction | 28 | 20 | 8 | 2.68 | 0.003 |
| GO:0046983~protein dimerization activity | 41 | 30 | 11 | 1.91 | 0.158 |
| GO:0030528~transcription regulator activity | 89 | 60 | 29 | 1.49 | 0.164 |
| GO:0005125~cytokine activity | 19 | 14 | 5 | 2.59 | 0.399 |
| GO:0008009~chemokine activity | 8 | 7 | 1 | 4.94 | 0.588 |
| GO:0042379~chemokine receptor binding | 8 | 7 | 1 | 4.64 | 0.914 |
| hsa04350:TGF-beta signaling pathway | 12 | 10 | 2 | 3.34 | 0.919 |
| hsa04621:NOD-like receptor signaling pathway | 9 | 7 | 2 | 3.91 | 1.012 |
| GO:0016564~transcription repressor activity | 26 | 23 | 3 | 2.08 | 1.151 |
| hsa04062:Chemokine signaling pathway | 17 | 10 | 7 | 2.33 | 1.874 |
| hsa05200:Pathways in cancer | 25 | 18 | 7 | 1.92 | 2.11 |
| GO:0003700~transcription factor activity | 58 | 38 | 20 | 1.5 | 2.625 |
| Database for Annotation, Visualization and Integrated Discovery. Significant pathways determined by false discovery rate (FDR) < 5%. GO (Gene Ontology), hsa (KEGG pathway). | | | | | |
