# Supplementary material for: Changes in Pre- and Post-Exercise Gene Expression among Patients with Chronic Kidney Disease and Kidney Transplant Recipients
Source: PLoS One. 2016 Aug 12;11(8):e0160327. doi: 10.1371/journal.pone.0160327 (PMC4982681; doi:10.1371/journal.pone.0160327)
Supplement: S2 Table — (DOCX) [file pone.0160327.s002.docx]

**Supplemental Table 2.** Gene ontology (GO) molecular function analysis by DAVID on genes after transplant that had at least a 1.5-fold greater expression before transplant

| **Term** | **Number** | **P Value** | **Genes** | **Fold Enrichment** | **False Discovery Rate** |
| --- | --- | --- | --- | --- | --- |
| GO:0003690~double-stranded DNA binding | 4 | 1.39E-04 | EGR1, KLF6, FOS, JUNB | 35.7 | 0.14 |
| GO:0043566~structure-specific DNA binding | 4 | 4.54E-04 | EGR1, KLF6, FOS, JUNB | 23.9 | 0.47 |
| GO:0003700~transcription factor activity | 6 | 0.002667 | EGR1, KLF6, FOS, EGR2, HEYL, JUNB | 5.3 | 2.71 |
| GO:0030528~transcription regulator activity | 7 | 0.003235 | EGR1, KLF6, FOS, EGR2, HEYL, WWTR1, JUNB | 4.0 | 3.28 |
| GO:0016563~transcription activator activity | 4 | 0.008779 | EGR1, KLF6, WWTR1, JUNB | 8.4 | 8.67 |
| GO:0046983~protein dimerization activity | 4 | 0.01867 | FOS, BDKRB2, WWTR1, JUNB | 6.4 | 17.63 |

Database for Annotation, Visualization and Integrated Discovery. 23 significant probes (P ≤ 0.05 and fold change > 1.5) that had at least a 1.5 fold greater response before transplant. Significant pathways determined by nominal P value < 0.05.
